# Detection and Sizing of Surface Flaws With a SQUID-Based Eddy Current Probe

**DOI:** 10.6028/jres.092.003

**Published:** 1987-02-01

**Authors:** J. C. Moulder, T. E. Capobianco

**Affiliations:** National Bureau of Standards, Boulder, CO 80303

**Keywords:** aluminum alloy, eddy current testing, flaw sizing, nondestructive evaluation, reflection probe, superconducting quantum interference device, surface flaw

## Abstract

In a new approach to eddy current detection and sizing of surface-breaking flaws, we have coupled a conventional reflection probe to a superconducting quantum interference device (SQUID) to produce an eddy current probe with increased sensitivity and signal to noise ratio. The reflection probe consists of an air-core excitation coil surrounding two counterwound ferite-core pickup coils connected in series. A room-temperature probe is inductively coupled to a SQUID, which operates in a liquid helium bath. The new probe was used to obtain flaw signals from a number of electrical-discharge machined slots in aluminum alloy 6061. Results indicated that by scanning the probe along the length of the flaw, the length could be determined from the extent of the flaw signal. The peak amplitude of the flaw signal was found to be proportional to the cross-sectional area of the flaw. Empirical calibration curves relating these quantities were used to invert successfully the experimental data obtained for the EDM slots.

## Introduction

The development of superconducting quantum interference devices (SQUIDs) two decades ago [1]^1^ introduced to the metrologist a new sensor of extraordinary sensitivity. The SQUID comprises a small superconducting inductance in series with a weak Josephson junction [2]. The rf impedance of the junction is a periodic function of the flux linking the series inductor, with a period of precisely one quantum of flux, *ϕ*_0_ = h/2e = 2.07 × 10^−15^ webers. If a feedback circuit is used to maintain a constant amount of flux linking the inductor in response to changes in the externally applied field, an error signal that is linear in flux results with wide dynamic range and a sensitivity of a fraction of a flux quantum [3]. The operating principles of such rf-biased devices are described in detail elsewhere [3–5]. The commercial availability of SQUIDs has led to their application to a wide variety of measurement problems: from magnetic-monopole detectors to magnetocardiography and magnetoencephalography. They have been used in magnetometers and gradiometers, for susceptibility measurements and noise thermometry, to name but a few applications.

One of the first applications of SQUIDS to measurement problems in nondestructive evaluation was the use of a SQUID magnetometer to measure and map the magnetic fields of eddy current probes [6,7]. The success of this application led to the development of a SQUID-based eddy current probe for flaw detection [8]. This instrument consisted of a reflection probe, operated at room temperature, coupled inductively to the SQUID, which is operated in a liquid helium bath (4K).

Our first studies with this probe characterized its responses to flaws by scanning the probe along or across several rectangular, electrical-discharged machined (EDM) slots in 6061 Al. We found that the magnitude of the flaw signal scaled linearly with cross-sectional area of the flaws (length times depth for these rectangular flaws). A linear response to flaw area has also been reported for electric-current perturbation (ECP) probes [9].

A linear response to flaw area suggests a particularly simple and direct method for determining the length and depth of surface-breaking cracks, the two parameters of most interest in a fracture-mechanics assessment of fitness for service. Measurements made by scanning the probe along the length of EDM slots [8] indicated that the length of the slot could be inferred from the “flaw profile” so produced, a procedure proposed for absolute probes by B. A. Auld et al. [10]. If the length and area of a flaw can be determined from the width and height of the flaw profile, and if the flaw shape is known, then it is quite simple to determine the flaw’s depth.

The objective of the present study was to evaluate the ability of the SQUID-based eddy current probe to detect and size surface-breaking flaws using the inversion method outlined above. A series of 16 EDM slots of different length, depth, and shape was machined in 6061 Al and scanned with the SQUID-based eddy current probe. Calibration curves relating the breadth and amplitude of the flaw signal to flaw length and cross-sectional area were derived from the experimental data. We then used these calibration curves to predict the size of the slots from the experimental data and compared the predictions with the known slot dimensions.

## Experiment

The eddy current probe used in these studies consists of a large (8.1-mm i.d.) air-core coil surrounding two smaller ferrite-core coils. The axes of all the coils are normal to the workpiece. The outer coil is driven with an oscillator to provide excitation; the flaw signal is taken from the two inner pickup coils connected in series opposing. Tuning slugs are located above each of the pickup coils to permit nulling of the signal in the absence of a flaw. The probe has been described in greater detail elsewhere [7,8].

**Figure 1:**
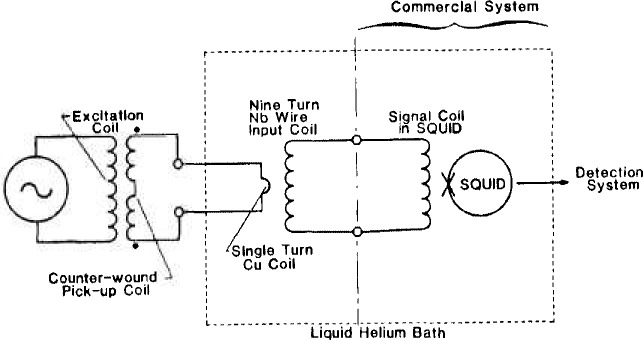
Schematic diagram of SQUID-based eddy current probe.

Connection of the eddy current probe to the SQUID is shown in figure 1. The room-temperature probe is connected to a single loop of copper wire in the liquid-helium Dewar through a shielded twisted pair. The eddy current signal is coupled to the SQUID inductively through the mutual inductance of the one-turn coil and a nine-turn Nb coil that is directly connected to the SQUID input terminals. The one-turn coil and the nine-turn coil are wound on separate coil forms; one coil is mounted on a translator so that the spacing (and strength of coupling) can be varied from outside the cryostat.

The SQUID and the detection electronics associated with it are commercial instruments. The output of the SQUID detector circuit was fed into a lock-in amplifier locked to the phase of the excitation voltage. In this way we could determine both the magnitude and phase of the flaw signals. The lock-in amplifier was interfaced to a laboratory computer through the IEEE-488 bus.

The eddy current probe was scanned over the flaws with a two-axis, computer-controlled positioner. Two types of scans were made: longitudinal and transverse. For longitudinal scans, one pickup coil is scanned along the length of the flaw with the other coil remaining to one side of the flaw. For transverse scans, the probe scans across the center of the flaw with the line connecting the two pickup coils at 90° to the flaw axis.

The EDM slots we used for this study are described in table 1. Four of the slots were semi-elliptical in cross section; the remainder were rectangular. They ranged in depth from 0.35 to 2.46 mm and in length from 3.69 to 7.08 mm. The widths of all the slots were about 0.2 mm. All the specimens were machined in 6061 Al alloy.

Measurements were performed under computer control in a step-and-measure mode. The probe was stepped along or across the flaws in 0.25-mm increments, remaining stationary at each point along the scan for a sequence of measurements of the flaw signal to be acquired by the computer from the lock-in amplifier. The operating frequency for the measurements reported here was 10 kHz. Previous measurements showed this to be the frequency of greatest sensitivity for the system [8]. Only results of longitudinal scans were used for signal inversion.

## Results and Discussion

Flaw profiles obtained by making longitudinal scans of the EDM slots in specimen NBS-5 are shown in figures 2a and 2b. Figure 2a shows the magnitude of the flaw signal and figure 2b shows the phase. There is a residual imbalance of about 50 mV in the magnitude of the SQUID output owing to our inability to perfectly balance the pickup of the two ferrite-core coils with the tuning slugs.

The double-peaked structure of the flaw profiles is characteristic of longitudinal scans; the two peaks occur when the pickup coil is near the ends of the flaw. In a way, the differential pickup coils are a magnetic field gradiometer; the emf developed across these coils is proportional to the gradient of the magnetic field normal to the surface. The double peaks indicate a concentration of eddy currents circulating around the flaw. The asymmetry of the two peaks appears to be related to probe construction, since the relative height of the two peaks reverses when the probe is rotated by 180°.

The structure of the phase flaw profiles shown in figure 2b is closely related to the structure of the magnitude profiles. Extrema in the phase curves occur at inflection points of the magnitude curves.

**Figure 3:**
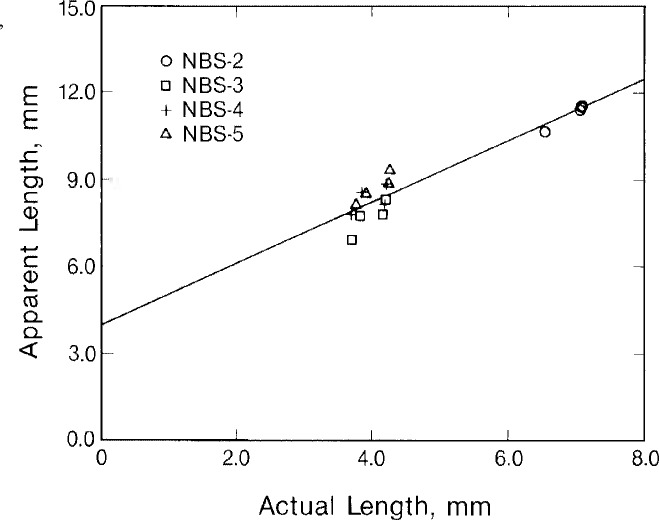
Correlation between flaw length and the width of the flaw signal obtained from a longitudinal flaw scan. Line is a least-squares fit to the data.

The characteristic flaw profiles shown in figure 2 may be used in a type of imaging to determine the length of the flaws. If we draw lines tangent to the flanks of the flaw profiles and determine the distance between intersections of these lines with the signal base line, we find this distance to be proportional to the actual flaw length as shown in figure 3 for all the specimens studied. The solid line in figure 3 is the result of a linear least-squares fit to the data.

**Figure 4:**
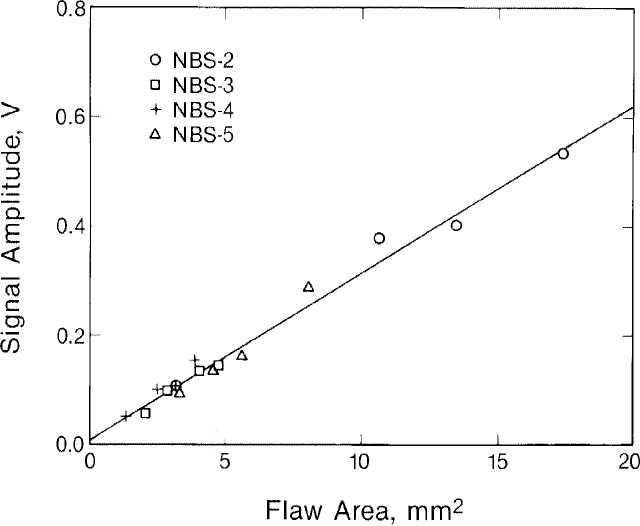
Relation between maximum flaw signal and cross sectional area of flaw. Line is a least-squares fit to the data.

The linear relationship between the peak amplitude of the flaw profile and cross-sectional area of the flaw is shown in figure 4 for the 16 flaws we studied. For this plot the amplitude of the larger peak was used. The solid line in figure 4 is a least-squares fit to the data. The results for the semi-elliptical flaws (NBS-3) can be seen to conform to the same response as the rectangular flaws.

**Figure 5:**
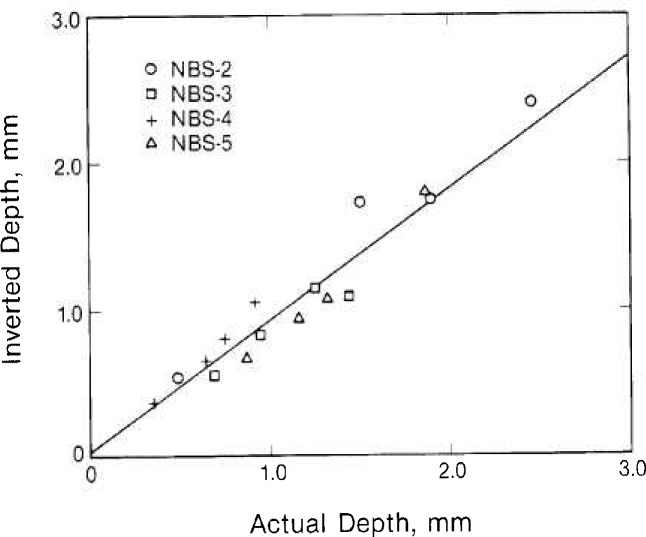
Flaw depth determined by inversion of eddy current flaw signals in relation to actual flaw depth. Line is a least-squares fit to the data.

If we now take the least-squares fits from figures 3 and 4 as calibration curves and use them to predict flaw lengths and depths from the experimental data, the results we obtain are shown in figure 5 and table 2. Table 2 lists the predicted and actual lengths and depths for the 16 EDM slots we studied. Figure 5 plots inverted depth against the actual depth. The solid line in figure 5 is a least-squares fit to the data. The fact that the slope of this line is slightly less than 45° indicates a tendency for the method to slightly underestimate the depth of flaws. On the whole, however, there is excellent agreement between predicted and actual flaw dimensions. In most cases the variance was less than 20 percent.

## Conclusions

A SQUID-based eddy current probe was used to obtain flaw signals from a number of EDM slots in Al 6061. The results indicated that the breadth of the flaw signal determined from the longitudinal scan of a flaw was proportional to the flaw length. The peak amplitude of the flaw signal was found to be proportional to cross-sectional area of the flaws. Empirical calibration curves relating these quantities were used to invert successfully the experimental data. The success of the simple inversion method used here indicates that this type of probe could be used to accurately size surface-breaking cracks with only a single calibration. Although the SQUID increased the magnitude of the response of the probe to a flaw by 80 dB, the main features of the flaw signal are related to the nature of the eddy current probe and not any special characteristics of the SQUID.

## Figures and Tables

**Figure 2a:**
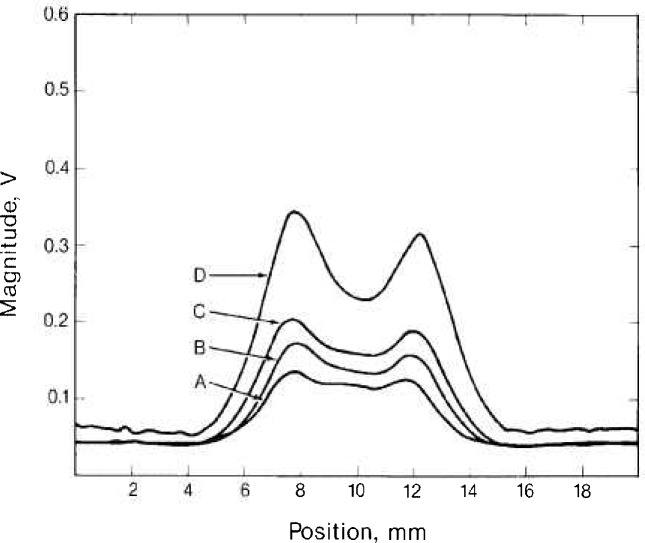
Magnitude of flaw signals from SQUID-based eddy current probe for longitudinal scans of four rectangular EDM slots in specimen NBS-5 (see table 1).

**Figure 2b:**
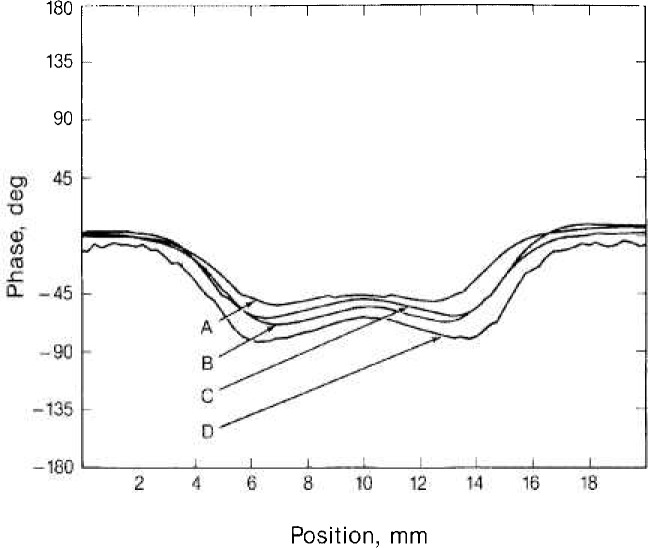
Phase of flaw signals for the same flaws shown in figure 2a.

**Table 1:** Flaw specimens used in experiments.

Specimen	Flaw Type	Length (mm)	Width (mm)	Depth (mm)
NBS-2A	R	6.54	0.24	0.49
NBS-2B	R	7.07	0.25	1.51
NBS-2C	R	7.08	0.32	1.90
NBS-2D	R	7.08	0.36	2.46
NBS-3A	S	3.70	0.19	0.70
NBS-3B	S	3.82	0.20	0.95
NBS-3C	s	4.15	0.22	1.25
NBS-3D	s	4.20	0.22	1.44
NBS-4A	R	3.69	0.17	0.35
NBS-4B	R	3.85	0.18	0.64
NBS-4C	R	4.16	0.19	0.75
NBS-4D	R	4.20	0.21	0.92
NBS-5A	R	3.75	0.24	0.88
NBS-5B	R	3.90	0.20	1.17
NBS-5C	R	4.24	0.18	1.32
NBS-5D	R	4.27	0.19	1.88

Flaw Types: S = semi-elliptical EDM notch, R = rectangular EDM notch.

**Table 2:** Results of inverting flaw measurements.

Specimen	Length (mm)		Depth (mm)	
	Inverted	Actual	Inverted	Actual
NBS-2A	6.28	6.54	0.52	0.49
NBS-2B	7.01	7.07	1.72	1.51
NBS-2C	7.10	7.08	1.75	1.90
NBS-2D	7.13	7.08	2.40	2.46
NBS-3A	2.75	3.70	0.55	0.70
NBS-3B	3.55	3.82	0.82	0.95
NBS-3C	3.57	4.15	1.15	1.25
NBS-3D	4.05	4.20	1.05	1.44
NBS-4A	3.57	3.69	0.37	0.35
NBS-4B	4.30	3.85	0.66	0.64
NBS-4C	3.91	4.16	0.80	0.75
NBS-4D	4.54	4.20	1.05	0.92
NBS-5A	3.91	3.75	0.68	0.88
NBS-5B	4.25	3.90	0.95	1.17
NBS-5C	4.59	4.24	1.08	1.32
NBS-5D	5.02	4.27	1.80	1.88

## Footnotes

1 Bracketed figures indicate literature references.
